# Prediction of Three-Dimensional Ground Reaction Forces in the Golf Swing Using Wearable Inertial Measurement Units and Biomimetic Deep Learning Models

**DOI:** 10.3390/biomimetics11030159

**Published:** 2026-02-27

**Authors:** Jiayun Li, Ruoyu Wei, Qiantong Xie, Changfa Wu, Yoon Hyuk Kim

**Affiliations:** 1 School of Social Sports, Tianjin University of Sport, Tianjin 301617, China; jiayunli829@gmail.com; 2 Department of Mechanical Engineering, Kyung Hee University, Yongin 17104, Republic of Korea; wry1997@khu.ac.kr; 3 College of Physical Education, Shenzhen University, Shenzhen 518060, China; qiantongxie@gmail.com; 4 School of Sport and Training, Tianjin University of Sport, Tianjin 301617, China; kinelens_changfawu@foxmail.com

**Keywords:** golf swing, biomechanics, wearable motion analysis, biomimetics, deep learning, inertial measurement units, ground reaction force

## Abstract

Ground reaction force (GRF) is essential for maintaining dynamic stability and generating power during the golf swing. Traditional GRF assessment relies on force plates, limiting measurement to laboratory environments and restricting evaluation of natural, field-based performance. Recent work has explored wearable inertial measurement units (IMUs) and data-driven models to estimate GRF during simple locomotor tasks, yet no study has examined whether coupled lower-limb kinematics can predict three-dimensional GRF during complex, high-speed movements such as the golf swing. This study collected bilateral hip, knee, and ankle joint angles from IMUs, along with 3D GRF data, to evaluate five biomimetic deep learning (DL) architectures across seven sensor configurations. The TCN-BiGRU model achieved the highest accuracy (R^2^ = 0.94 ± 0.02, MRE = 0.044 ± 0.01, NRMSE = 0.064 ± 0.01) among the architectures evaluated in this study, effectively capturing both local and long-range temporal dependencies in human movement. The full bilateral lower-limb configuration yielded the best overall performance, whereas using only the lead leg provided a cost-efficient alternative with minimal loss of accuracy. Among the GRF components, the vertical direction showed the greatest predictive reliability. These findings demonstrate the feasibility and potential of kinematic–force modeling and support the development of wearable, field-ready systems for GRF estimation in dynamic sports environments.

## 1. Introduction

A golf swing is a highly coordinated, full-body movement that demands precise biomechanical control from the athlete [1]. Among various biomechanical variables, ground reaction forces (GRF) are a critical determinant of swing power output, movement efficiency, postural stability, and injury risk [2]. Accurate quantification of GRFs during the swing provides essential insight into lower limb loading patterns and their contribution to force transmission along the kinetic chain [3,4]. However, traditional GRF measurements rely on fixed force plates, which confine data collection to controlled laboratory environments [5,6]. Since golf swings predominantly occur in natural outdoor settings, this constraint highlights the need for portable and wearable alternatives for measuring GRFs during golf performance.

Inertial measurement units (IMUs) represent a lightweight, wearable, and cost-effective alternative to force plates, offering the ability to collect high-frequency motion data in real-world environments [7,8]. Through the measurement of linear accelerations and angular velocities, IMUs enable the indirect estimation of GRFs based on Newtonian mechanics, especially when placed on distal segments close to the ground. Recently, many studies have explored the feasibility of estimating GRFs using IMU-derived features across various movements, including walking, running, jumping, and daily activities [9,10,11,12]. For instance, Alcantara et al. attached an IMU to the sacrum and used an LSTM network to predict vertical GRFs during treadmill running, achieving an NRMSE of 0.16 [13]. Similarly, Inai and Takabayashi combined IMU signals from the shank and sacrum with a multilayer perceptron (MLP), obtaining vertical GRF predictions with NRMSE as low as 0.27 [14]. These findings indicate that IMUs, combined with deep learning (DL) models, can provide a practical approach for estimating GRFs without direct force measurements.

However, many existing methods rely on simplified modeling techniques such as linear regression or fixed feature extraction, which may be insufficient to capture the complex, nonlinear dynamics between multi-axis IMU signals and GRFs [15,16]. Furthermore, prior studies have primarily focused on relatively repetitive, planar movements—such as gait, straight-line running, or vertical jumps—that exhibit more predictable force patterns [15,17,18]. In contrast, the golf swing involves rapid axial rotation, asymmetric weight transfer, and temporally precise loading patterns, presenting unique challenges for accurate GRF estimation [19,20]. Recently, Mori and Kwon employed a Bi-LSTM model to estimate 3D GRFs, yet their reliance on laboratory-based optical motion capture restricts the method’s utility in field settings [21]. Consequently, the application of IMU-based DL frameworks to complex, high-speed rotational movements such as the golf swing has yet to be systematically investigated.

DL can be considered a biomimetic method because its multilayer neural architecture is inspired by the information-processing principles of biological neural systems, enabling the model to learn complex kinematic–force relationships from data [22,23]. In golf swing analysis, DL algorithms have been increasingly used to capture nonlinear coordination patterns, temporal sequencing, and multi-segment interactions that conventional modeling approaches cannot represent [24,25]. From a biomimetic perspective, the five models evaluated in this study emulate different aspects of biological computation: MLP captures simplified neural processing, CNN extracts spatially organized motion features, GRU and LSTM-based models mimic temporal memory in motor control, and hybrid TCN-BiGRU integrates both local pattern extraction and long-range temporal dependency, resembling hierarchical sensorimotor processing [26,27,28]. Therefore, comparing these architectures provides insight into which bio-inspired computational strategy best models the natural kinematic–force coupling in the golf swing.

Given the biomechanical complexity of the golf swing, the accurate estimation of GRFs is essential for understanding swing mechanics and injury mechanisms. These characteristics highlight the need to examine the feasibility of predicting GRFs from wearable IMUs using DL approaches during the golf swing. Therefore, this study aims to systematically compare the performance of various DL architectures—including feedforward, convolutional, recurrent, and hybrid models—for predicting three-dimensional GRFs during golf swings based on lower-limb IMU data. Furthermore, we investigate how different sensor placement configurations influence prediction accuracy, with the goal of identifying optimal model structures and sensor placement configurations.

## 2. Materials and Methods

### 2.1. Participants

24 males and 24 females’ healthy professional golfers (23.2 ± 1.2 years; height: 175.3 ± 3.1 cm; body mass: 80.1 ± 8.0 kg; handicap: 1.9 ± 1.5) participated in this study. All participants were right-handed and reported no history of musculoskeletal disorders, chronic pain, or serious injuries within the previous six months. Written informed consent was obtained from all participants, and the study protocol was approved by the Research and Ethics Committee of the School of Physical Education, Tianjin University of Sport.

### 2.2. Experimental Protocol and Data Collection

**Figure 1:**
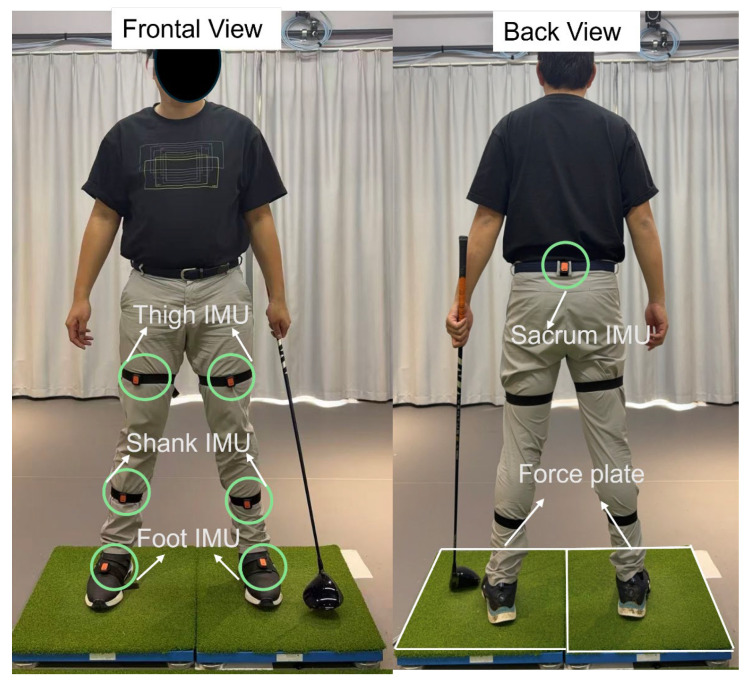
IMU placement location and force plate set up.

Two three-dimensional portable force plates (Type 9260AA6, Kistler Instrumente AG, Winterthur, Switzerland; sampling frequency = 2400 Hz) were used to collect ground reaction force (GRF) data. The force plates provide high measurement accuracy and reliability, with linearity < ±0.5% of full-scale output (FSO), hysteresis < 0.5% FSO, and inter-channel crosstalk < ±2.5%. Each participant placed one foot on each plate, allowing independent recording of left- and right-foot GRFs throughout the golf swing. IMU system (Xsens Dot, Movella Inc., Henderson, NV, USA, weight: 11.2 g, size: 36.3 mm × 30.4 mm × 10.8 mm, sampling frequency = 60 Hz) was employed, with seven sensors mounted on the feet, shanks, thighs, and pelvis (Figure 1). Sensors were secured with elastic straps to minimize soft-tissue motion during high-speed rotation. Before data collection, participants performed 1–3 familiarization swings to adjust to the setup and force-plate positions. Each participant then completed 10 full golf swings at a self-selected stance and natural rhythm, using the same Driver club (Callaway Golf, Carlsbad, CA, USA). IMU data were used to compute three-dimensional joint angles of the hip, knee, and ankle and were temporally synchronized with GRF data from the force plates. Both GRF and joint-angle signals were processed using a fourth-order Butterworth low-pass filter, with cutoff frequencies of 6 Hz for GRF and 12 Hz for joint angles. To maintain statistical independence between samples, the filtered data were time-normalized to 0–100% of the swing phase using cubic-spline interpolation for each trial independently.

### 2.3. DL Models

#### 2.3.1. Five DL Models

The five neural network architectures were selected to represent a spectrum of sequence modeling capabilities. The MLP serves as a baseline for nonlinear signal integration. The CNN was included for its ability to extract local spatial features from multi-sensor arrays. GRU and LSTM architectures were employed to model temporal dependencies and sequence memory. Finally, the hybrid TCN-BiGRU was implemented to combine the local receptive field advantages of temporal convolutions with the long-range dependency capture of bidirectional recurrent units, aiming to robustly model the complex dynamics of the golf swing.

#### 2.3.2. Model Training

We employed seven joint-angle input configurations (Table 1) in combination with five DL models (Table 2) to estimate 3D-GRFs during the golf swing. The seven input configurations (Set A–G) represent different joint selection strategies, incorporating unilateral or bilateral hip, knee, and ankle joint angles (computed across all three anatomical planes, totaling three dimensions per joint). These configurations were designed to systematically examine how input dimensionality and the number of required sensors influence prediction performance.

All models were trained using a unified training strategy: a batch size of 16, 25 training epochs, and parameter optimization via the Adam optimizer. The mean squared error (MSE) was adopted as the loss function to minimize the discrepancy between the predicted and measured GRFs. To prevent overfitting, the validation loss was continuously monitored during training, and an early stopping criterion was applied when no further improvement was observed.

The key structural parameters and layer configurations of each model are summarized in Table 2. Specifically, the MLP consisted of three fully connected layers; the CNN model included three convolutional layers for extracting local temporal features; and the GRU model stacked two GRU layers to capture temporal dependencies. However, previous studies have noted that standard RNNs and CNNs may be limited in capturing long-range dependencies in non-periodic, high-speed movements like golf swings [10,11,21,29,30]. To address these limitations, the CNN-LSTM combined convolution-based feature extraction with LSTM units for long-range sequence modeling, and the TCN-BiGRU incorporated three TCN blocks together with two BiGRU layers to simultaneously learn multi-scale temporal dynamics and bidirectional temporal information.

#### 2.3.3. Model Evaluation

The predictive performance of each model was quantitatively assessed using five statistical indices: the coefficient of determination (R^2^), the mean absolute error (MAE), the mean relative error (MRE), the root mean square error (RMSE), and the normalized root mean square error (NRMSE). Together, these metrics characterize accuracy, relative deviation, and overall goodness of fit between the predicted and measured GRFs. All statistical analyses and error computations were performed in Python (version 3.12; Python Software Foundation, Wilmington, DE, USA). To assess the generalization ability of the proposed model and strictly prevent data leakage, a subject-level 10-fold cross-validation procedure was implemented. The 48 participants were partitioned into 10 folds: the first 8 folds contained 5 participants each, while the remaining 2 folds contained 4 participants each. Crucially, all trial data belonging to a specific participant were exclusively assigned to the same fold, ensuring that the model was evaluated solely by unseen participants not present in the training set. To statistically compare model performance, fold-level metrics were used for paired comparisons between each baseline model and the proposed TCN-BiGRU model. Paired Wilcoxon signed-rank tests with Holm correction were applied, and statistical significance was defined as *p* < 0.05.

## 3. Results

### 3.1. Training and Validation Loss

**Figure 2:**
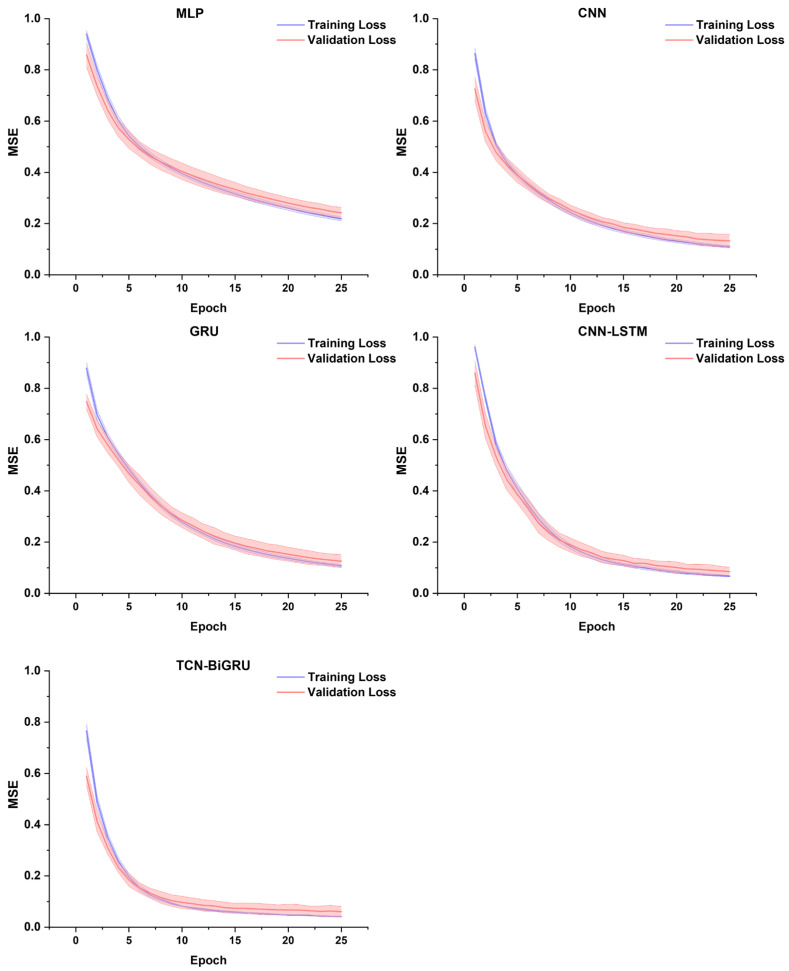
Training loss and validation loss curve for five models in each epoch. Solid lines represent the mean loss, and shaded areas indicate the standard deviation (SD) across the validation folds.

All five DL models demonstrated stable convergence over the 25 training epochs, with both training and validation losses decreasing consistently. Among the models, TCN-BiGRU achieved the lowest final validation loss (≈0.12), followed by CNN-LSTM (≈0.15) and GRU (≈0.17). The CNN also exhibited effective convergence, reaching a final validation loss of roughly 0.18, while the MLP showed the slowest learning dynamics, stabilizing at a comparatively higher validation loss of around 0.22. The standard deviation bands across the 10 folds were narrow for all models, especially after epoch 10, suggesting stable and repeatable learning behavior (Figure 2).

### 3.2. Comparison of Model Prediction Performance

**Figure 3:**
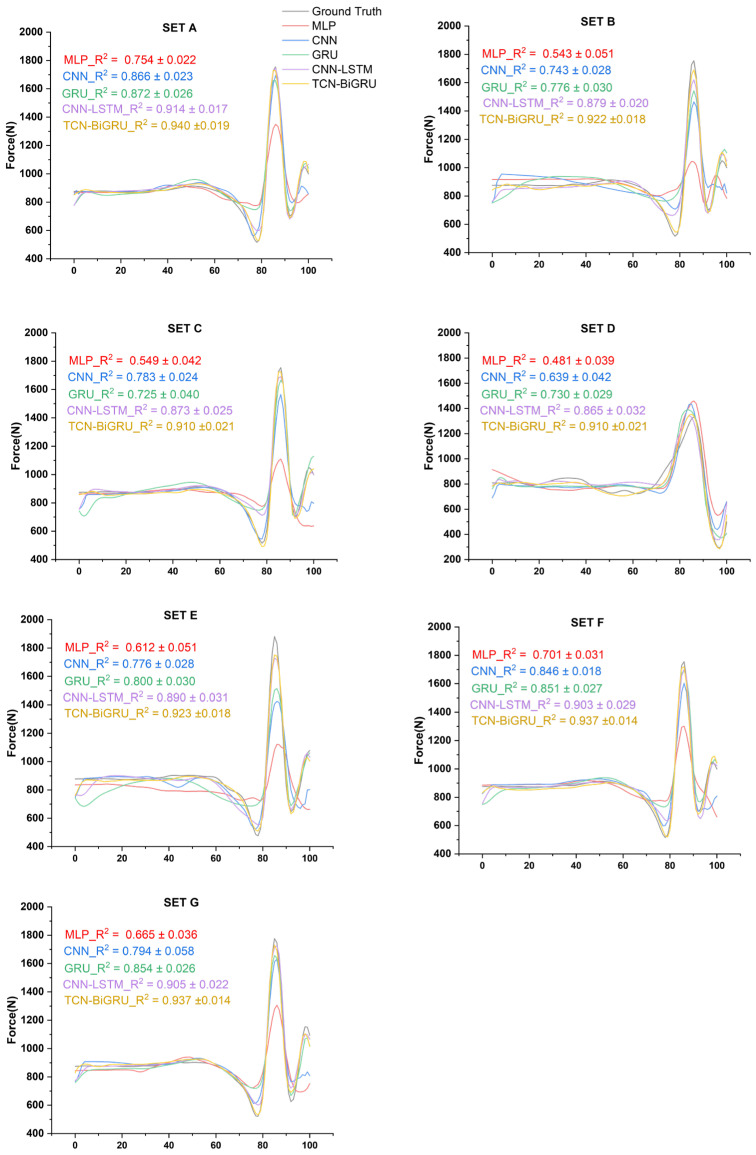
Comparison of predicted resultant ground reaction force waveforms and correlation coefficients (R^2^) using five DL models across seven sensor placement sets. The resultant force was calculated as the magnitude of the three-dimensional force vector $F=\sqrt[{}]{{F_{x}}^{2}+{F_{y}}^{2}+{F_{z}}^{2}}$.

**Figure 4:**
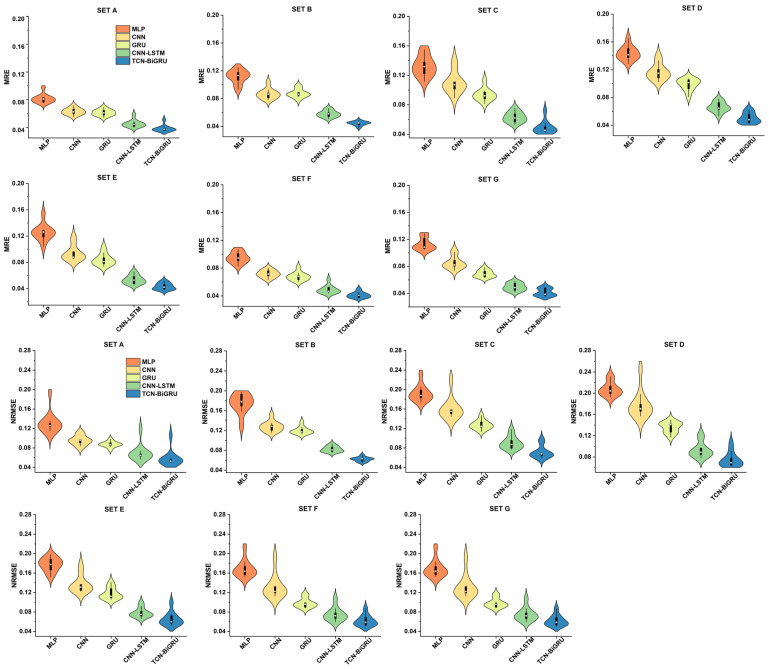
Comparison of mean relative error (MRE) and normalized root mean squared error (NRMSE) in ground reaction force predictions using DL models across seven sensor placement sets. The violin plots show data density (width), median (white dot), and interquartile range (black bar).

**Figure 5:**
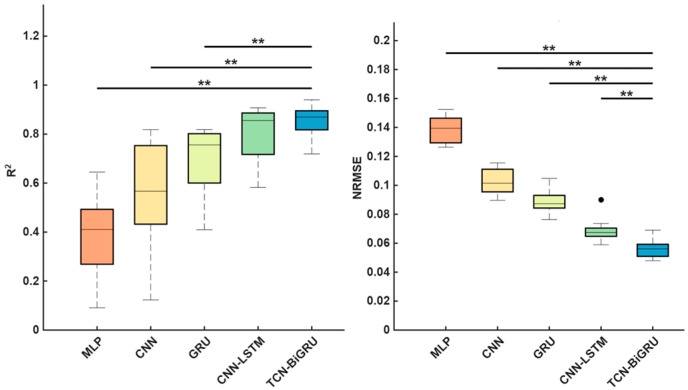
Statistical comparison of R^2^ and normalized root mean squared error (NRMSE) of five deep learning architectures for ground reaction force predictions. Differences were considered statistically significant at *p* < 0.05 (** *p* < 0.01). The black dot represents an outlier. The boxplots show the median (central line), interquartile range (box), and non-outlier data range.

The R^2^ values of MLP, CNN, GRU, CNN-LSTM, and TCN-BiGRU across seven IMU placement sets are shown in Figure 3, while the corresponding MRE and NRMSE values for these models are presented in Figure 4. In all sets, the models’ performance followed the order: MLP < CNN < GRU < CNN-LSTM < TCN-BiGRU, with TCN-BiGRU exhibiting the highest R^2^ and the lowest MRE and NRMSE. In contrast, MLP demonstrated the lowest R^2^ along with the highest MRE and NRMSE. As shown in Figure 5, statistical analysis further confirmed that the TCN-BiGRU model significantly outperformed all baseline models (*p* < 0.05). To provide quantitative statistical evidence, Table 3 summarizes the median performance, percentage improvement, and Holm-adjusted *p*-values from paired Wilcoxon signed-rank tests comparing TCN-BiGRU with the strongest baseline model (CNN-LSTM). The results indicate consistent and statistically significant reductions in NRMSE (14.2–25.2%, *p* < 0.01) across all seven placement sets, along with significant improvements in R^2^ (up to 16.4%) in the majority of configurations, further confirming the robustness of the proposed mode.

### 3.3. Effect of Sensor Placement on Model Performance

**Figure 6:**
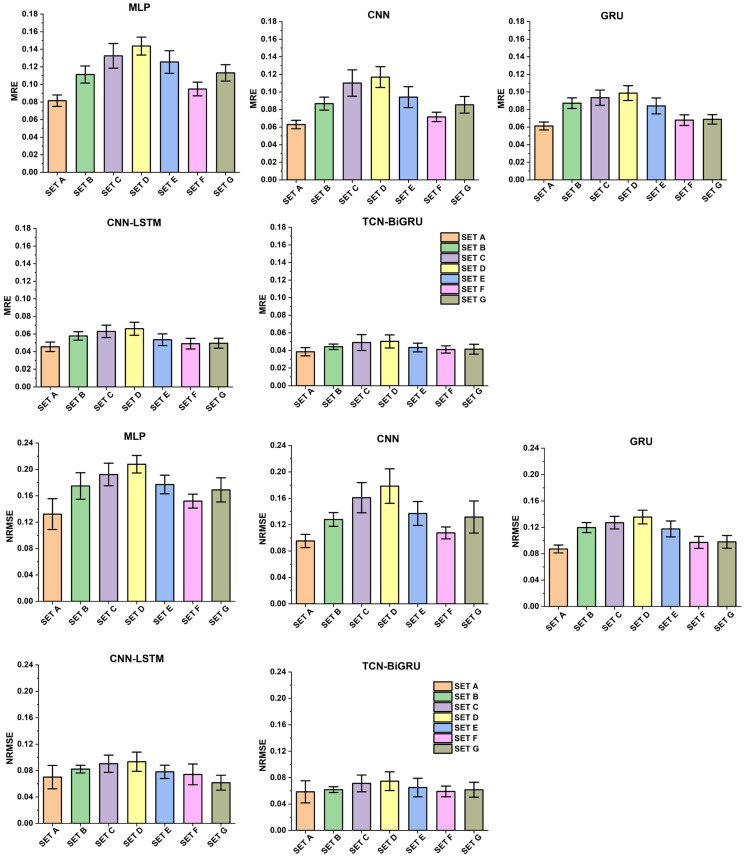
Mean and standard deviation (SD) of Mean relative error (MRE) and normalized root mean squared error (NRMSE) of ground reaction force predictions across seven sensor placement sets using five DL models.

The comparison of MRE and NRMSE across seven sets and five models (MLP, CNN, GRU, CNN-LSTM, and TCN-BiGRU) is shown in Figure 6. The error values, from highest to lowest, are ranked as follows: Set D > Set C > Set B > Set E > Set G > Set A > Set F.

### 3.4. Comparative Evaluation Across GRF Directions

The comparison of GRF prediction results across the three axes (X, Y, Z) is shown in Table 4. The Z-axis exhibited higher R^2^ values compared to the X and Y axes. The MRE and NRMSE values for the Z-axis were notably smaller than those for the X and Y axes, whereas the MAE and NRMSE for the Z-axis were significantly larger.

## 4. Discussion

This study developed and evaluated a DL framework for predicting 3D GRFs during the golf swing using IMU-based joint kinematics. We compared multiple neural network architectures and sensor configuration schemes to determine optimal prediction performance. Three key findings emerged. First, the TCN-BiGRU achieved the highest accuracy (R^2^ = 0.94 ± 0.02; MRE = 0.044 ± 0.01; NRMSE = 0.064 ± 0.01), reflecting its strong ability to capture both local and long-range temporal dependencies. Second, prediction accuracy differed notably across joint-angle configurations, with the full bilateral set (Set A) and the lead-side configuration (Set E) outperforming single-joint inputs. Third, the vertical (Z-axis) component was consistently predicted most accurately, exceeding the anterior–posterior (Y-axis) and medio–lateral (X-axis).

The improved performance of the TCN-BiGRU arises from its hybrid design that integrates temporal convolutional networks (TCNs) with bidirectional GRUs [31]. The TCN module expands the temporal receptive field through dilated convolutions and residual connections, enabling efficient extraction of local and multi-scale temporal features from IMU-derived joint kinematics. The BiGRU then models long-range, bidirectional temporal dependencies, providing a more complete representation of the sequential coordination between the backswing and downswing—an aspect that the unidirectional LSTM cannot fully capture. Similarly, Mori and Kwon showed that bidirectional LSTMs effectively handle the golf swing’s complex, non-periodic phases [21]. In contrast, while CNN-LSTM models have shown strong performance in repetitive, rhythmic tasks such as walking and running, their assumptions about periodicity are less suited to the highly non-periodic, rapidly rotating, and asymmetrically loaded nature of the golf swing [10,29,30]. Together, the multi-timescale filtering of the TCN and the bidirectional temporal integration of the BiGRU contribute to the TCN-BiGRU’s improved predictive performance and stability relative to the baseline models when modeling the complex force-generation dynamics of the golf swing (Table 5).

From a biomimetic perspective, the superior performance of the TCN-BiGRU can be attributed to its structural alignment with biological motor control mechanisms. Specifically, the failure of the MLP and standard CNNs to achieve comparable accuracy suggests that the golf swing cannot be modeled as a sequence of isolated states or purely local spatial patterns. Instead, the success of the TCN-BiGRU aligns with the biological concept of the ‘kinetic chain,’ where forces are sequentially transferred across segments [33,34]. The TCN component effectively decodes these hierarchical motor synergies, like how the nervous system organizes complex movements into modular primitives [35,36]. Furthermore, the BiGRU’s bidirectional processing mirrors the ‘internal models’ (forward and inverse models) utilized by the cerebellum, which integrate past sensory states with future movement anticipation to regulate stability [37]. The model’s ability to minimize error implies that it successfully emulated this biological strategy, effectively bridging the gap between discrete kinematic inputs and continuous, dynamic force outputs in a way that simpler bio-inspired models (like MLP or unidirectional RNNs) could not (Table 6).

Model performance was strongly influenced by both the number and anatomical location of the joint angles. The bilateral multi-joint configuration (Set A) yielded the highest accuracy, as combining proximal and distal kinematics provides more comprehensive information for GRF prediction [38,39]. However, this configuration requires many sensors across multiple segments, limiting its practicality. Therefore, identifying reduced-sensor setups that still achieve high accuracy remains an important objective in GRF prediction research [40]. Our results showed that when only four IMUs were used to provide bilateral data for a single joint, predictive performance decreased from the ankle to the knee and then to the hip, with ankle-based inputs performing best. This trend can be explained by the ankle’s substantial contribution to vertical loading and propulsion during the golf swing [41]. Yılmazgün et al. similarly reported that joint kinematics captured closer to the point of ground contact provide more accurate and relevant information for GRF prediction [11]. Importantly, the lead-side configuration (Set E) achieved accuracy comparable to the full bilateral arrangement, despite using only four sensors. This finding indicates that the lead leg alone provides sufficient kinetic representation for estimating 3D GRFs during the golf swing [3,19,42]. Among all reduced-sensor configurations, lead-side placement outperformed bilateral single-joint inputs, suggesting it offers the most efficient balance between accuracy and practicality.

Prediction accuracy showed a clear direction-dependent pattern, with the vertical component achieving the highest accuracy, followed by the medio–lateral and anterior–posterior components. This hierarchy is biomechanically reasonable for the golf swing. The vertical GRF exhibits relatively consistent loading patterns across swings, as it primarily reflects weight transfer and lead-leg bracing during impact [43,44]. In contrast, the medio–lateral component is more sensitive to individual differences in swing style and rotational strategy, leading to moderate variability and slightly lower accuracy [42,45]. The anterior–posterior GRF component exhibited the lowest prediction accuracy. This may be attributed to both the relatively small magnitude of the anterior–posterior forces and their heightened sensitivity to subtle variations in braking and propulsion timing among individual golfers. Additionally, from a measurement perspective, anterior–posterior and medio–lateral GRF components are generally much smaller than the vertical component, resulting in a lower signal-to-noise ratio that may impair model performance [46]. These findings suggest that GRF components dominated by large, consistent loading patterns are more readily captured by DL models, whereas components characterized by smaller amplitudes or greater inter-individual variability pose greater challenges [47,48].

These direction-dependent GRF patterns also emphasize the need for models with different capacities to capture both stable and variable force features. The observed performance disparities among the architectures highlight the importance of structural complexity in capturing these mechanics. While simpler models like MLP and CNN lacked the temporal integration required for continuous coordination, the TCN-BiGRU demonstrated superior accuracy. By integrating TCN layers—which capture long-range dependencies similar to auditory processing—with bidirectional recurrent units akin to hippocampal memory, the model effectively extracts both local features and global temporal dynamics. This architecture aligns well with the natural hierarchical sensorimotor processing required for the complex, non-linear coupling of the golf swing

There are several limitations in this study. First, the GRF reference data were collected using force plates under controlled indoor conditions, which limits ecological validity. Specifically, the distinct shoe-surface interaction may alter GRF patterns, thereby affecting the model’s field generalization to natural outdoor environments [49]. Second, the participant cohort consisted exclusively of healthy professional golfers to ensure high kinematic consistency for baseline validation. However, this homogeneity limits the model’s generalizability to populations with greater variability, such as amateur golfers, older adults, or individuals with musculoskeletal pathologies [47,50]. Third, soft-tissue artifacts, magnetometer disturbances, and sensor orientation drift could degrade IMU signal quality, particularly during the rapid rotational phases of the downswing. Additionally, the relatively low IMU sampling rate (60 Hz) may be insufficient to fully capture high-frequency transient dynamics, particularly around club impact [51,52]. Finally, the proposed DL models are purely data-driven and do not incorporate explicit biomechanical constraints. While such models can capture complex nonlinear mapping relationships, they may lack interpretability and may not extrapolate well beyond the training domain [53,54]. Future research should investigate hybrid physics-informed architectures and larger, multi-speed datasets to enhance model generalizability and real-world applicability.

## 5. Conclusions

This study developed a DL framework for estimating three-dimensional GRFs during the golf swing using IMU-based lower-limb kinematics. Among all architectures, the TCN-BiGRU achieved the highest accuracy due to its ability to capture both short-term kinematic fluctuations and long-range temporal dependencies. Sensor-placement analysis showed that a lead-side hip–ankle configuration provides accuracy comparable to a full bilateral setup, suggesting that a compact sensor arrangement is sufficient for practical, field-based GRF estimation. From a biomimetic standpoint, the model’s multi-timescale convolutions and bidirectional recurrent pathways parallel cerebellar–cortical information processing, explaining its superior performance in representing the coordination of the golf swing. Direction-specific analyses further indicated that vertical GRFs were most accurately predicted, followed by medio-lateral and anterior–posterior components, consistent with their biomechanical variability and signal characteristics. Overall, these findings demonstrate the feasibility of combining wearable sensors with DL for non-laboratory GRF estimation and highlight the potential for portable, real-time systems for swing assessment and injury prevention. Future work should expand datasets across broader skill levels and environments and explore physics-informed modeling to strengthen robustness and generalization.

## Figures and Tables

**Table 1:** Seven joint-angle input configurations, the number of input parameters, sensor placement locations, and the required number of sensors. Joint angles were considered across all three anatomical planes: sagittal, frontal, and transverse.

Set	Number of Input Parameters	Included Joints	Number of IMUs
Set A	18	Bilateral hip, knee, and ankle joints in three anatomical plane	7
Set B	6	Bilateral ankle joints in three anatomical plane	4
Set C	6	Bilateral knee joints in three anatomical plane	4
Set D	6	Bilateral hip joints in three anatomical plane	3
Set E	9	Lead-leg hip, knee, and ankle joints in three anatomical plane	4
Set F	12	Bilateral ankle and knee joints in three anatomical plane	6
Set G	12	Bilateral hip and knee joints in three anatomical plane	5

**Table 2:** Optimal parameter architecture of the five deep learning (DL) model.

Model	Batch Size	Hidden/Feature Dim	Epochs	Stacked Layers
MLP	16	64	25	3 fully connected layers
CNN	16	CNN Chanels = 32 → d_model = 64	25	3 convolutional layers
GRU	16	d_model = 64	25	2 GRU layers
CNN-LSTM	16	CNN Chanels = 32 → d_model = 64 + LSTM d_model = 64	25	2 convolutional layers + 2 LSTM layers
TCN-BiGRU	16	TCN d_model = 64 + GRU d_model = 64	25	3 TCN blocks + 2 GRU layers

**Table 3:** Median performance and statistical comparison between CNN-LSTM and TCN-BiGRU models across seven IMU placement sets. Statistical significance was assessed using paired Wilcoxon signed-rank tests with Holm correction (*n* = 10 folds, *p* < 0.05).

NRMSE				R^2^			
CNN-LSTM	TCN-BiGRU	Reduction (%)	*p* (Holm)	CNN-LSTM	TCN-BiGRU	Increase (%)	*p* (Holm)
0.044	0.037	16.4	0.0078 (**)	0.873	0.901	3.12	0.3359
0.06	0.045	22.2	0.0078 (**)	0.802	0.863	7.72	0.0078 (**)
0.064	0.049	25.2	0.0078 (**)	0.708	0.824	16.44	0.0391 (*)
0.066	0.054	18.7	0.0078 (**)	0.736	0.823	11.80	0.0371 (*)
0.052	0.041	20.6	0.0078 (**)	0.823	0.884	7.46	0.0078 (**)
0.049	0.042	16.9	0.0078 (**)	0.856	0.869	1.61	0.0645
0.048	0.04	14.2	0.0078 (**)	0.867	0.858	−1.02	0.2520

Note: The *p*-value of 0.0078 corresponds to the lowest possible value in the Wilcoxon signed-rank test for the given sample size, indicating that the proposed TCN-BiGRU consistently outperformed the baseline across all validated folds in these specific comparisons. * *p* < 0.05, ** *p* < 0.01.

**Table 4:** Mean and standard deviation of the coefficient of determination (R^2^), mean absolute error (MAE), mean relative error (MRE), root mean square error (RMSE), and normalized RMSE (NRMSE) for the five models in predicting GRFs across the anterior–posterior X-axis (anterior–posterior), Y-axis (medio–lateral), and Z-axis (vertical).

		R^2^			MAE			MRE			RMSE			NRMSE		
		X-Axis	Y-Axis	Z-Axis	X-Axis	Y-Axis	Z-Axis	X-Axis	Y-Axis	Z-Axis	X-Axis	Y-Axis	Z-Axis	X-Axis	Y-Axis	Z-Axis
MLP	Mean	0.61	0.6	0.63	15.06	29.71	86.13	1.88	1.28	0.11	23.05	40.72	129.43	2.87	1.76	0.17
	SD	0.13	0.08	0.12	2.43	3.3	16.22	0.37	0.2	0.02	3.94	4.76	20.74	0.6	0.28	0.03
CNN	Mean	0.77	0.76	0.77	11.71	22.93	67.34	1.46	0.99	0.09	17.64	31.21	100.72	2.2	1.35	0.13
	SD	0.08	0.08	0.12	1.86	3.51	14.73	0.29	0.19	0.02	3.43	4.98	23.24	0.49	0.26	0.03
GRU	Mean	0.81	0.77	0.84	11.32	22.93	60.27	1.41	0.99	0.08	15.91	30.62	84.01	1.98	1.32	0.11
	SD	0.06	0.06	0.05	1.5	2.88	10.84	0.26	0.16	0.01	2.36	4.15	13.73	0.39	0.22	0.02
CNN-LSTM	Mean	0.89	0.87	0.92	8.92	16.99	41.22	1.11	0.73	0.05	12.53	23.27	60.42	1.56	1.01	0.08
	SD	0.03	0.04	0.03	0.98	2.13	6.62	0.19	0.13	0.01	1.69	3.26	10.72	0.3	0.18	0.02
TCN-BiGRU	Mean	0.92	0.92	0.95	7.35	13.06	32.99	0.92	0.56	0.04	10.52	18.5	48.45	1.31	0.8	0.06
	SD	0.02	0.03	0.02	0.61	1.47	4.74	0.14	0.08	0.01	1.18	2.86	9.07	0.22	0.14	0.01

**Table 5:** Comparison with external state-of-the-art studies on DL-based ground reaction force prediction.

Study	Task	Input Modality	Output	Model	Best Performance
Lee et al. (2020) [32]	Walking	Single IMU (sacrum)	3D GRF	ANN/RF	NRMSE = 6.7% (vertical GRF)
Alcantara et al. (2022) [13]	Running (up/downhill)	Sacrum & shoe accelerometers	Normal GRF (vertical only)	RNN/LSTM	RMSE = 0.16 ± 0.04 BW; rRMSE = 6.4 ± 1.5%
Carter et al. (2024) [16]	Treadmill running	Wearable IMUs + pressure insoles	Vertical GRF	LSTM	rRMSE = 0.8–8.8%
Yılmazgün et al. (2025) [11]	Multiple tasks	IMUs (various configurations)	3D GRF	CNN	rRMSE = 6.2% (vertical GRF, best configuration)
Chen et al. (2025) [10]	Running (multi-speed)	IMU-derived joint angles	Vertical GRF	CNN-xLSTM	R^2^ = 0.909; rMSE = 0.061
Mori et al. (2025) [21]	Golf swing	Motion capture kinematics + force plate	Vertical GRF	Bi-LSTM	ICC = 0.983
This study	Golf swing	IMU-based joint kinematics	3D GRF	TCN-BiGRU	R^2^ = 0.94; NRMSE = 0.064; MRE = 0.044

**Table 6:** Qualitative comparison of five neural network architectures in golf swing analysis.

Model	Key Mechanism	Applicability to Golf Swing	Primary Limitation
MLP	Global mapping; no explicit sequence modeling.	Low	Treats movement as static frames; ignores kinetic chain continuity.
CNN	Convolutional extraction of local spatial/temporal features.	Moderate	Limited receptive field; fails to capture long-range dependencies.
GRU/LSTM	Unidirectional recurrent modeling of temporal sequences.	Moderate	Lacks future context; struggles with asymmetric backswing-downswing dynamics.
CNN-LSTM	Hybrid: Local feature extraction + sequential modeling.	High	Implicitly assumes rhythmic periodicity; less ideal for discrete, rapid motions.
TCN-BiGRU	Multi-scale dilated convolutions + Bidirectional integration.	Optimal	Higher computational complexity compared to baseline models.

## Data Availability

The datasets during the current study are available from the corresponding author on reasonable request.

## References

[1] Hume P.A., Keogh J., Reid D. (2005). The role of biomechanics in maximising distance and accuracy of golf shots. Sports Med..

[2] McNitt-Gray J.L., Munaretto J., Zaferiou A., Requejo P.S., Flashner H. (2013). Regulation of reaction forces during the golf swing. Sports Biomech..

[3] Ancillao A., Tedesco S., Barton J., O’Flynn B. (2018). Indirect Measurement of Ground Reaction Forces and Moments by Means of Wearable Inertial Sensors: A Systematic Review. Sensors.

[4] Purevsuren T., Kwon M.S., Park W.M., Kim K., Jang S.H., Lim Y.T., Kim Y.H. (2017). Fatigue injury risk in anterior cruciate ligament of target side knee during golf swing. J. Biomech..

[5] Purevsuren T., Khuyagbaatar B., Kim K., Kim Y.H. (2018). Investigation of Knee Joint Forces and Moments during Short-Track Speed Skating Using Wearable Motion Analysis System. Int. J. Precis. Eng. Man..

[6] TKhurelbaatar T., Kim K., Lee S., Kim Y.H. (2015). Consistent accuracy in whole-body joint kinetics during gait using wearable inertial motion sensors and in-shoe pressure sensors. Gait Posture.

[7] Lim H., Kim B., Park S. (2020). Prediction of Lower Limb Kinetics and Kinematics during Walking by a Single IMU on the Lower Back Using Machine Learning. Sensors.

[8] Hossain M.S.B., Guo Z.S., Choi H. (2023). Estimation of Lower Extremity Joint Moments and 3D Ground Reaction Forces Using IMU Sensors in Multiple Walking Conditions: A Deep Learning Approach. IEEE J. Biomed. Health Inform..

[9] Liu X.Z., Zhang X.L., Zhang B., Zhou B., He Z.X., Liu T. (2024). An IMU-Based Ground Reaction Force Estimation Method and Its Application in Walking Balance Assessment. IEEE Trans. Neural Syst. Rehabil. Eng..

[10] Chen T., Xu D., Zhou Z., Zhou H., Shao S., Gu Y. (2025). Prediction of Vertical Ground Reaction Forces Under Different Running Speeds: Integration of Wearable IMU with CNN-xLSTM. Sensors.

[11] Yılmazgün B., Weber J., Stein T., Sell S., Stetter B.J. (2025). Predicting 3D ground reaction forces across various movement tasks: A convolutional neural network study comparing different inertial measurement unit configurations. J. Biomech..

[12] Kerns J.A., Zwart A.S., Perez P.S., Gurchiek R.D., McBride J.M. (2023). Effect of IMU location on estimation of vertical ground reaction force during jumping. Front. Bioeng. Biotechnol..

[13] Alcantara R.S., Edwards W.B., Millet G.Y., Grabowski A.M. (2022). Predicting continuous ground reaction forces from accelerometers during uphill and downhill running: A recurrent neural network solution. PeerJ.

[14] Inai T., Takabayashi T. (2022). Estimation of lower-limb sagittal joint moments during gait using vertical ground reaction force. J. Biomech..

[15] Baker L.M., Yawar A., Lieberman D.E., Walsh C.J. (2024). Predicting overstriding with wearable IMUs during treadmill and overground running. Sci. Rep..

[16] Carter J., Chen X., Cazzola D., Trewartha G., Preatoni E. (2024). Consumer-priced wearable sensors combined with deep learning can be used to accurately predict ground reaction forces during various treadmill running conditions. PeerJ.

[17] Koshio T., Haraguchi N., Takahashi T., Hara Y., Hase K. (2024). Estimation of Ground Reaction Forces during Sports Movements by Sensor Fusion from Inertial Measurement Units with 3D Forward Dynamics Model. Sensors.

[18] Mohamed Refai M.I., van Beijnum B.F., Buurke J.H., Veltink P.H. (2020). Portable Gait Lab: Estimating Over-Ground 3D Ground Reaction Forces Using Only a Pelvis IMU. Sensors.

[19] Najafi B., Lee-Eng J., Wrobel J.S., Goebel R. (2015). Estimation of Center of Mass Trajectory using Wearable Sensors during Golf Swing. J. Sports Sci. Med..

[20] Lynn S.K., Wang J., Schmitt A.C., Barnes C.L. (2023). Lower Body Joint Moments during the Golf Swing in Older Adults: Comparison to Other Activities of Daily Living. J. Sports Sci. Med..

[21] Mori K. (2025). Estimation of the Ground Reaction Forces During Golf Swing Using Recurrent Neural Networks. ISBS Proc. Arch..

[22] Kriegeskorte N., Golan T. (2019). Neural network models and deep learning. Curr. Biol..

[23] Zhang J., Zhao Y., Shone F., Li Z., Frangi A.F., Xie S.Q., Zhang Z.Q. (2023). Physics-Informed Deep Learning for Musculoskeletal Modeling: Predicting Muscle Forces and Joint Kinematics From Surface EMG. IEEE Trans. Neural. Syst. Rehabil. Eng..

[24] Jiao L., Bie R., Wu H., Wei Y., Ma J., Umek A., Kos A. (2018). Golf swing classification with multiple deep convolutional neural networks. Int. J. Distrib. Sens. Netw..

[25] Kim M., Park S. (2020). Golf Swing Segmentation from a Single IMU Using Machine Learning. Sensors.

[26] Hofmann M., Becker M.F.P., Tetzlaff C., Mader P. (2025). Concept transfer of synaptic diversity from biological to artificial neural networks. Nat. Commun..

[27] Cohen Y., Engel T.A., Langdon C., Lindsay G.W., Ott T., Peters M.A.K., Shine J.M., Breton-Provencher V., Ramaswamy S. (2022). Recent Advances at the Interface of Neuroscience and Artificial Neural Networks. J. Neurosci..

[28] Lynn H.M., Pan S.B., Kim P. (2019). A Deep Bidirectional GRU Network Model for Biometric Electrocardiogram Classification Based on Recurrent Neural Networks. IEEE Access.

[29] Hwang S., Kwon N., Lee D., Kim J., Yang S., Youn I., Moon H.J., Sung J.K., Han S. (2025). A Multimodal Fatigue Detection System Using sEMG and IMU Signals with a Hybrid CNN-LSTM-Attention Model. Sensors.

[30] Jaramillo I.E., Jeong J.G., Lopez P.R., Lee C.-H., Kang D.-Y., Ha T.-J., Oh J.-H., Jung H., Lee J.H., Lee W.H. (2022). Real-Time Human Activity Recognition with IMU and Encoder Sensors in Wearable Exoskeleton Robot via Deep Learning Networks. Sensors.

[31] He J.L., Wang J.H., Lo C.M., Jiang Z. (2025). Human Activity Recognition via Attention-Augmented TCN-BiGRU Fusion. Sensors.

[32] Lee M., Park S. (2020). Estimation of Three-Dimensional Lower Limb Kinetics Data during Walking Using Machine Learning from a Single IMU Attached to the Sacrum. Sensors.

[33] Putnam C.A. (1993). Sequential motions of body segments in striking and throwing skills: Descriptions and explanations. J. Biomech..

[34] Halilaj E., Rajagopal A., Fiterau M., Hicks J.L., Hastie T.J., Delp S.L. (2018). Machine learning in human movement biomechanics: Best practices, common pitfalls, and new opportunities. J. Biomech..

[35] d’Avella A., Saltiel P., Bizzi E. (2003). Combinations of muscle synergies in the construction of a natural motor behavior. Nat. Neurosci..

[36] Merel J., Botvinick M., Wayne G. (2019). Hierarchical motor control in mammals and machines. Nat. Commun..

[37] Vyas S., Golub M.D., Sussillo D., Shenoy K.V. (2020). Computation Through Neural Population Dynamics. Annu. Rev. Neurosci..

[38] Weygers I., Kok M., Konings M., Hallez H., De Vroey H., Claeys K. (2020). Inertial Sensor-Based Lower Limb Joint Kinematics: A Methodological Systematic Review. Sensors.

[39] Hernandez V., Dadkhah D., Babakeshizadeh V., Kulic D. (2021). Lower body kinematics estimation from wearable sensors for walking and running: A deep learning approach. Gait Posture.

[40] Liang W., Wang F., Fan A., Zhao W., Yao W., Yang P. (2023). Deep-learning model for the prediction of lower-limb joint moments using single inertial measurement unit during different locomotive activities. Biomed. Signal. Process. Control.

[41] Bourgain M., Rouch P., Rouillon O., Thoreux P., Sauret C. (2022). Golf Swing Biomechanics: A Systematic Review and Methodological Recommendations for Kinematics. Sports.

[42] You X., Xu Y., Liang M., Baker J.S., Gu Y. (2023). The Relationship between Ground Reaction Forces, Foot Positions and Type of Clubs Used in Golf: A Systematic Review and Meta-Analysis. Appl. Sci..

[43] Miller J.D., Cabarkapa D., Miller A.J., Frazer L.L., Templin T.N., Eliason T.D., Garretson S.K., Fry A.C., Berkland C.J. (2023). Novel 3D Force Sensors for a Cost-Effective 3D Force Plate for Biomechanical Analysis. Sensors.

[44] Bourgain M., Sauret C., Rouillon O., Thoreux P., Rouch P. (2017). Contribution of vertical and horizontal components of ground reaction forces on global motor moment during a golf swing: A preliminary study. Comput. Methods Biomech. Biomed. Eng..

[45] Joo S.B., Oh S.E., Mun J.H. (2016). Improving the ground reaction force prediction accuracy using one-axis plantar pressure: Expansion of input variable for neural network. J. Biomech..

[46] Kammoun A., Ravier P., Buttelli O. (2024). Comparison of the Accuracy of Ground Reaction Force Component Estimation between Supervised Machine Learning and Deep Learning Methods Using Pressure Insoles. Sensors.

[47] Kim J., Kim K.C., Tack G., Choi J.S. (2025). Estimation of 3D Ground Reaction Force and 2D Center of Pressure Using Deep Learning and Load Cells Across Various Gait Conditions. Sensors.

[48] Johnson W.R., Mian A., Robinson M.A., Verheul J., Lloyd D.G., Alderson J.A. (2021). Multidimensional Ground Reaction Forces and Moments From Wearable Sensor Accelerations via Deep Learning. IEEE Trans. Biomed. Eng..

[49] Smith T., Ditroilo M. (2023). Force plate coverings significantly affect measurement of ground reaction forces. PLoS ONE.

[50] Dindorf C., Dully J., Konradi J., Wolf C., Becker S., Simon S., Huthwelker J., Werthmann F., Kniepert J., Drees P. (2024). Enhancing biomechanical machine learning with limited data: Generating realistic synthetic posture data using generative artificial intelligence. Front. Bioeng. Biotechnol..

[51] Rong R., Kuo C. (2024). Dynamic Soft Tissue Artifacts during Impulsive Loads: Measurement Errors Vary With Wearable Inertial Measurement Unit Sensor Design. IEEE Trans. Biomed. Eng..

[52] Kim M., Park S. (2024). Enhancing accuracy and convenience of golf swing tracking with a wrist-worn single inertial sensor. Sci. Rep..

[53] Ahmed S.F., Alam M.S.B., Hassan M., Rozbu M.R., Ishtiak T., Rafa N., Mofijur M., Shawkat Ali A.B.M., Gandomi A.H. (2023). Deep learning modelling techniques: Current progress, applications, advantages, and challenges. Artif. Intell. Rev..

[54] Gavrishchaka V., Senyukova O., Koepke M. (2019). Synergy of physics-based reasoning and machine learning in biomedical applications: Towards unlimited deep learning with limited data. Adv. Phys. X.

